# Iodine monitoring models contribute to avoid adverse birth outcomes related more than adequate iodine intake

**DOI:** 10.1186/s12884-021-03936-w

**Published:** 2021-06-28

**Authors:** Jinju Dong, Shouyan Liu, Lingyun Wang, Xingjian Zhou, Qinghong Zhou, Congli Liu, Jingrui Zhu, Weilan Yuan, Wang-yang Xu, Jie Deng

**Affiliations:** 1 Department of Gynaecology and Obstetrics, Xiangyang No.1 People’s Hospital, Hubei University of Medcine, Xiangyang, 441000 China; 2 Department of Obstetrics, Pingdingshan No.1 People’s Hospital, Pingdingshan, 467000 China; 3 Biotecan Medical Diagnostics Co., Ltd, Zhangjiang Center for Translational Medicine, Shanghai, 201204 China; 4 Singlera Genomics (Shanghai) Ltd, Shanghai, 201318 China

**Keywords:** More than adequate iodine, Monitoring model, Macrosomia

## Abstract

**Background:**

Iodine plays an important role in pregnancy. How to maintain adequate iodine intake amongst pregnant women in each trimester of pregnancy to prevent adverse birth outcomes in central China is a challenge for clinical practice.

**Methods:**

870 pregnant women and their infants were enrolled in the study. Urinary iodine concentration (UIC) was measured using an inductively coupled plasma mass spectrometry (ICP-MS). Maternal and newborn information were obtained during follow-up. Multinomial logistic regression models were established.

**Results:**

Median UIC of pregnant women was 172 ± 135 μg/L which is currently considered to be sufficient. Multivitamin supplements containing iodine, iodized salt intake and frequent milk intake were significantly associated with higher UIC. Multivariate logistic regression analysis showed that multivitamin supplements containing iodine and milk consumption were risk factors for more than adequate iodine (UIC ≥ 250 μg/L). Iodine-rich diet was significantly related to heavier birthweight, larger head circumference and longer femur length of the newborns while more than adequate iodine intake (UIC ≥ 250 μg/L) was a risk factor for macrosomia. Logistic regression models based on potential risk factors involving iodine containing supplements and iodine-rich diet were established to predict and screen pregnant women with high risk of more than adequate iodine intake among local pregnant women in different trimesters and guide them to supplement iodine reasonably to prevent the risk.

**Conclusions:**

Multivitamin supplements containing iodine and milk consumption were risk factors for maternal UIC ≥ 250 μg/L which was a risk factor for macrosomia. Iodine monitoring models were established to provide guidance for pregnant women to reduce the risk of more than adequate iodine intake, thereby contributing to reduce the risk of having a macrosomia.

**Supplementary Information:**

The online version contains supplementary material available at 10.1186/s12884-021-03936-w.

## Introduction

Iodine is an essential component for the synthesis of thyroid hormones (THs) which are important for the growth and maturation of fetus and the development of brain [1]. Sufficient iodine intake and TH levels are essential to prevent the birth abnormality of the offspring [2, 3]. Previous studies have shown that the increased risk of abortus, stillbirth, low birthweight infants, macrosomia, preterm delivery, neurological damage, and intellectual impairment are closely related to maternal iodine deficiency [4–7]. Therefore, adequate iodine intake during pregnancy is an important measure to ensure the health of pregnant women and the growth and maturation of fetus.

More than 90% of the body’s iodine sources are food, and most of the iodine is excreted through urine [8]. Therefore, UIC acts a good index to reflect the population iodine status during pregnancy [9]. Chinese government has implemented a universal salt iodization (USI) policy since 1996. In the beginning, the Ministry of Health stipulated that the standard of iodine content in edible salt was as follows: the iodine concentration of iodized salt (calculated by iodine ion) was processed to 50 mg/kg, not less than 40 mg/kg when it was manufactured, not less than 30 mg/kg when it was sold, and not less than 20 mg/kg when it was used by users. Adjusted continuously, in 2011, the Ministry of Health issued the “iodine content of edible salt”, which stipulated that the average level of iodine content in edible salt should be lowered to 20 mg/kg ~ 30 mg/kg, which can be divided into 20 mg/kg, 25 mg/kg and 30 mg/kg. One or two kinds of iodized salt can be supplied according to the iodine nutrition level of people in the province. China has nearly wiped out iodine deficiency disease (IDD) in the past two decades. China is currently considered to be iodine sufficient [10]. Although iodine levels in many provinces have been greatly improved, the harm of iodine excess to pregnant women and fetus is also attracting attention. A study from Wuhan, another large city in Hubei Province, pointed out that maternal iodine deficiency and excess during pregnancy have adverse effects on fetal growth and development, and put forward the necessity of monitoring the iodine status of pregnant women to ensure normal iodine nutrition during pregnancy [11]. Research reports in Henan Province showed that UIC of reproductive-age women and pregnant women were more than adequate in recent years and women with UIC above 249 μg/L was significantly correlated with increased risk of abnormal pregnancy outcomes [12, 13].

The present study aimed to assess iodine status in central China and explore the effect of maternal UIC on neonatal outcomes. Because the best indicator to evaluate iodine status is still controversial, and considering the influence of iodized salt and iodine-rich diet on iodine status and reducing the influence of excessive iodine intake on newborns, we finally constructed a model in each trimester to predict and screen pregnant women with high risk of more than adequate iodine intake based on potential risk factors involving iodine containing supplements, iodized salt and iodine-rich diet and guided them to take iodine reasonably, thus helping to reduce the risk of macrosomia related more than adequate iodine intake.

## Methods

### Participants

In this study, 1023 pregnant women were recruited from the obstetrics department of the No.1 People’s Hospital of Xiangyang City, Hubei Province and the No.1 People’s Hospital of Pingdingshan City, Henan Province, from December 2017 to December 2019. The exclusion criteria were as follows: (1) a history of primary and metastatic tumor; (2) a history of severe cardiovascular and cerebrovascular disease, such as stroke, myocardial infarction, myocarditis, hereditary heart diseases and coronary heart disease; (3) a history of drug abuse; (4) a history of thyroidectomy; (5) infant mortality or major defects; (6) loss of follow-up; (7) lack of laboratory examination or questionnaire. Finally, 870 pregnant women and their newborns were included in the study. Participants filled in the clinical questionnaire including age, gestational weeks, pre-pregnancy body mass index (BMI), dietary habits, iodine supplement, use of iodized salt and medical records during pregnancy. Passed after three experts reviewed the clarity and understanding of the questionnaire and agreed to all items, data was collected by trained nurses via face-to-face interview. The construct validity and reliability of the questionnaire were analyzed. The Kaiser-Meyer-Olkin value (KMO) coefficient was 0.66. Bartlett’s test yielded *P* <  0.0001 representing the quality of the sample. The reliability coefficient (alpha) of the questionnaire was 0.85, which was considered acceptable. Gestational weeks were divided into: First trimester, 1–12 weeks; Second trimester, 13–27 weeks; Third trimester, 28–40 weeks. BMI (kg/m^2^) were divided into four subgroups including below 18.5, 18.5–23.9, 24–27.9, and above 28 according to China standard (National Health and Family Planning Commission of the People’s Republic of China, 2013). Influence of diet, including seafood (three groups: no seafood consumption; occasional: 1–2 meal containing seafood per week on average; frequent: at least 3 meal containing seafood per week), egg (three groups: no egg consumption; occasional: 1–2 eggs per week on average; frequent: at least 3 eggs per week), cow’ milk and yogurt (three groups: no consumption; occasional: 1–500 ml per week on average; frequent: at least 500 ml per week), were recorded according to the above classification. Xiangyang and Pingdingshan Municipal governments both chose iodized salt with 25 mg/kg iodine. Every pregnant woman was required to provide the package (including nutrition table) of household salt for cooking. The photo template of the package of household salt was shown in Supplementary Fig. 1. Pregnant women with iodized salt for cooking is defined as the use of iodized salt, without iodized salt for cooking is defined as no iodized salt use. The multivitamin tablets prescribed by the doctor for some pregnant women with vitamin deficiency contain 50 μg iodine / per tablet. Pregnant women take one or two tablets daily (50 μg or 100 μg of iodine intake) according to the doctor’s advice. Whether each pregnant woman used multivitamin tablets, or if they bought them privately, and how much iodine the tablets contain were recorded. Pregnant women taking multivitamin supplements containing iodine are defined as iodine supplements, pregnant women taking multivitamin supplements without iodine or pregnant women not taking multivitamin supplements are defined as non-iodine supplements. However, in this study, all participants received iodine supplements from multivitamin supplements and no pregnant women was given pure iodine supplements, so we descripted it as multivitamin containing iodine instead of iodine supplements. Newborn information including birth date, sex, length, weight, femur length and head circumference were obtained from medical records. The study has been performed in accordance with the Declaration of Helsinki and was approved by the Research Ethics Committee (201702170b) of Hubei Medical College. Informed consents were provided for all participants.

### Urine iodine measurement

Participants were asked to void their bladders in the morning, then started a 24-h urine sampling, and 1.5 ml of urine from a random urine was taken during the 24-h sampling process. Urine samples were placed at 4 °C and detected in duplicate by inductively coupled plasma mass spectrometry (ICP-MS) (Agilent Technologies, Inc., Tokyo, Japan) as described in a previous study within 8 h after receiving [14]. In short, 100 μl urine samples were extracted with ammonium hydroxide solution. Tellurium was used as an internal standard. Self-made quality control material was used in every run to ensure the accuracy of the method. The average concentration of the quality control sample is 140 ± 4.5 μg/L. Coefficient of variation (CV) was 3.19%. Blank solution was routinely prepared and tested to monitor potential iodine cross contamination in urine samples. Participate in the External Quality Assessment (EQA) run by the National Health Commission of the People’s Republic of China twice 1 year. A recent “Successful” participation certificate of EQA issued by the National Health Commission for the ICP/MS method in December 2020 was attached as Supplementary Fig. 2. The iodine status of pregnant women is graded according to the WHO/ICCIDD reference, UIC < 150 μg/L is defined as deficiency, UIC of 150–250 μg/L is defined as adequate, UIC of 250–500 μg/L is defined as more than adequate and UIC ≥ 500 μg/L is defined as excess [15].

#### Multinomial logistic regression model

In the machine learning framework, the logistic regression models of the score were calculated using the variables (clinical risk factors) to predict risk. The logistic score according to the logistic regression equation can be obtained via the following formula:

Predicted risk probability = $\frac{{\mathrm{e}}^{\left(\upbeta 0+\sum \upbeta \mathrm{iXi}\right)}}{1+{\mathrm{e}}^{\left(\upbeta 0+\sum \upbeta \mathrm{iXi}\right)}}$ *β*0, the constant of the logistic regression formula. *Βi*, the coefficient of the variable *Xi*. Multinomial logistic regression is an extension of ordinary binary logistic regression analysis, in which classification dependent outcomes have more than two. The results were structured into three outcomes: iodine deficiency, adequate iodine and more than adequate iodine. Multinomial logistic regression models were selected to predict the probability of each outcome in different (three) trimester. The predictive values of outcomes were evaluated by the receiver operating characteristic (ROC) curve.

### Statistical analysis

Statistical analyses were conducted by using Python software (Version 3.6) (https://www.python.org). Python is an interpretive and general-purpose computer programming language which was designed by Guido van Rossum in the late 1980s. It has a dynamic type of system and emphasizes readability and rapid prototyping. Python keeps the second position in the TIOBE index (https://www.tiobe.com/tiobe-index/). SciPy. stat subpackage in Python was used to data processing and statistics. Continuous variables were shown as mean/median ± standard deviation (SD), and categorical variables as frequency or percentage. The association between UIC and clinical characteristics, and the association between UIC and infant characteristics were analyzed by using ANOVA or Kruskal-Wallis H test. The Sklearn package in Python was applied to construct the model and calculate the area under the curve (AUC) of the ROC. *P* <  0.05 was set as statistically difference.

## Results

### Iodine status among pregnant women in the Central China

The median UIC was 172 ± 135 μg/L among the pregnant women. About 30.5% of the pregnant women (265/870) were in the adequate UIC range (150–249 μg/L). Nearly 32.3% (281/870) showed a UIC of 250 μg/L or greater. On the contrary, 37.2% (324/870) of participants had a UIC < 150 μg/L (Supplementary Fig. 3). Actually, 14.3% of the pregnant women took multivitamin supplement containing iodine during pregnancy. About 90.7% of the pregnant women used iodized salt in this cohort. Macrosomia (weight > 4000 g) accounted for 4% of all infants (Table 1). More than 70% of pregnant women in this study were in the second trimester and had higher iodine levels than those in the first and third trimesters. Multivitamin supplement with iodine and frequent milk intake was associated with higher UIC (*P* <  0.0001). Iodized salt intake significantly elevated UIC compared with people who never eat iodized salt (*P* <  0.0001). Frequent seafood intake was associated with higher UIC, although not statistically significant (Supplementary Table 1).

**Table 1:** Baseline characteristics for total participants

Characteristics	n (%)	Mean ± SD
Total pregnancy women	870	
Age, years		28.4 ± 4.0
0–25	181 (20.8)	
26 ~ 35	643 (73.9)	
> 35	46 (5.3)	
Gestational week		16.6 ± 6.9
first trimester	178 (20.5)	
second trimester	628 (72.2)	
third trimester	64 (7.4)	
Pre-pregnancy BMI, kg/m^2^		22.7 ± 3.7
< 18.5	63 (7.2)	
18.5 ~ 24	569 (65.4)	
24.1 ~ 28.9	175 (20.1)	
≥ 29	63 (7.2)	
Multivitamin with iodine		
Yes	124 (14.3)	
No	746 (85.7)	
Iodized salt		
Yes	789 (90.7)	
No	81 (9.3)	
Sea food (fish, crab, shrimp)		
Frequent	135 (15.5)	
Occasional	667 (76.7)	
None	68 (7.8)	
Cow’ milk		
Frequent	412 (47.4)	
Occasional	397 (45.6)	
None	61 (7.0)	
Yogurt		
Frequent	144 (16.6)	
Occasional	521 (59.9)	
None	205 (23.6)	
Egg		
Frequent	347 (39.9)	
Occasional	478 (54.9)	
None	45 (5.2)	
Total infants	870	
Infants gender		
Male	468 (53.79)	
Female	402 (46.21)	
Infants weight (g)		3393.3 ± 294.4
< 4000	834 (95.86)	
≥ 4000	36 (4.14)	
Infants length (cm)		50.0 ± 1.7
Femur length (cm)		7.3 ± 0.3
Head circumference (cm)		33.6 ± 1.3

*SD* standard deviation

*BMI* body mass index

*UIC* urinary iodine concentration

### Maternal diet and neonatal characteristics

Multivitamin supplements containing iodine, iodized salt intake, and frequent consumption of seafood, milk and yogurt was significantly associated with a heavier birthweight. Iodized salt intake was significantly related to the larger head circumference of the newborns. Iodized salt and frequent seafood intake were associated with longer femur length (Table 2). No significant correlation was observed between neonatal characteristics and maternal age and pre-pregnancy BMI.

**Table 2:** Correlation between neonatal characteristics and maternal clinical factors

	Birth height (cm)	*P*	Birth weight (g)	*P*	Femur length (cm)	*P*	Head circumference (cm)	*P*
Multivitamin with iodine								
Yes	50 (49, 51)	0.231	3420 (3298, 3600)	0.001*	7.3 (7.1, 7.5)	0.996	33.5 (33.0, 34.5)	0.455
No	50 (49, 51)		3383 (3200, 3550)		7.3 (7.1, 7.5)		33.5 (33.0, 34.2)	
Drinker								
Yes	50 (49, 51)	0.705	3440 (3250, 3600)	0.078	7.3 (7.1, 7.4)	0.292	33.5 (33.0, 34.4)	0.771
No	50 (49, 51)		3400 (3200, 3550)		7.3 (7.1, 7.5)		33.5 (33.0, 34.2)	
Sea food								
Frequent	50 (49, 51)	0.964	3450 (3200, 3600)	0.015*	7.4 (7.2, 7.6)	0.034*	33.5 (33.0, 34.2)	0.290
Occasional	50 (49, 51)		3400 (3200, 3520)		7.3 (7.1, 7.5)		33.6 (33.1, 34.3)	
None	50 (49, 51)		3420 (3250, 3600)		7.3 (7.0, 7.5)		33.5 (32.8, 34.1)	
Egg								
Frequent	50 (49, 51)	0.440	3400 (3200, 3560)	0.550	7.3 (7.1, 7.5)	0.100	33.5 (33.0, 34.4)	0.536
Occasional	50 (49, 51)		3400 (3200, 3543)		7.3 (7.1, 7.5)		33.5 (33.0, 34.2)	
None	50 (50, 51)		3450 (3300, 3560)		7.5 (7.2, 7.6)		33.7 (33.2, 34.2)	
Cow’ milk								
Frequent	50 (49, 51)	0.365	3420 (3208, 3600)	< 0.001*	7.3 (7.1, 7.5)	0.594	33.6 (33.1, 34.3)	0.094
Occasional	50 (49, 51)		3400 (3200, 3520)		7.3 (7.1, 7.5)		33.5 (33.0, 34.2)	
None	50 (49, 51)		3300 (3150, 3500)		7.2 (7.0, 7.5)		33.5 (33.0, 33.9)	
Yogurt								
Frequent	50 (50, 51)	0.061	3450 (3250, 3625)	0.037*	7.3 (7.2, 7.5)	0.710	33.5 (33.0, 34.1)	0.521
Occasional	50 (49, 51)		3400 (3200, 3520)		7.3 (7.1, 7.5)		33.5 (33.0, 34.2)	
None	50 (49, 51)		3320 (3200, 3550)		7.3 (7.1, 7.5)		33.6 (33.2, 34.3)	
Iodized salt								
Yes	50 (49, 51)	0.868	3400 (3200, 3560)	0.002*	7.3 (7.1, 7.5)	< 0.001*	33.6 (33.0, 34.3)	< 0.001*
No	50 (49, 51)		3340 (3152, 3450)		7.2 (7.0, 7.3)		33.4 (32.9, 33.6)	

Data presented as mean (interquartile range, IQR)

*Statistically significant difference set at *P* < 0.05, Kruskal-Wallis H test

### Risk factors for exceeding adequate iodine intake

Clinical factors, such as multivitamin supplement with iodine and dietary intake are considered risk factors for iodine excess. In this study, more than adequate iodine (UIC ≥ 250 μg/L) was used as response variable, and risk factors as explanatory variables in the multivariate logistic regression. We found that multivitamin (containing iodine) supplements and milk intake were risk factors after additional adjustment for other risk factors (Supplementary Table 2).

### Maternal iodine status and macrosomia risk

Significant difference was found in birthweight among different UIC groups. Compared with the pregnant women in the UIC < 250 μg/L group, the birthweight in the UIC ≥ 250 μg/L group was heavier (*P* <  0.05). The femur length in the UIC of 250–499 μg/L group was longer than that < 150 μg/L group (*P* <  0.05). In the group with UIC < 150 μg/L, the infant head circumference was smaller than other groups (*P* < 0.05) (Supplementary Table 3). Multivariate logistic regression was used to evaluate maternal UIC and macrosomia risk. More than adequate (UIC ≥ 250 μg/L) and iodine excess (UIC ≥ 500 μg/L) during pregnancy acted as risk factors for macrosomia regardless of gestational weeks (Table 3) while iodine deficiency was not a risk factor for macrosomia (Supplementary Table 4).

**Table 3:** Multivariate logistic regression analysis on macrosomia risk

UIC (μg/L)	Macrosomia OR (95% CI)	*P*-value
0–149	Ref	
150–249	1.40 (0.53, 3.68)	0.496
250–499	2.94 (1.25, 6.94)	0.014^*^
≥500	2.66 (1.51, 7.78)	0.043^*^

Data presented as odds ratio (OR) (95% confidence interval (CI))

*Statistically significant difference set at *P* < 0.05

### Monitor models for guide iodine supplement to prevent more than adequate iodine intake

**Fig. 1:**
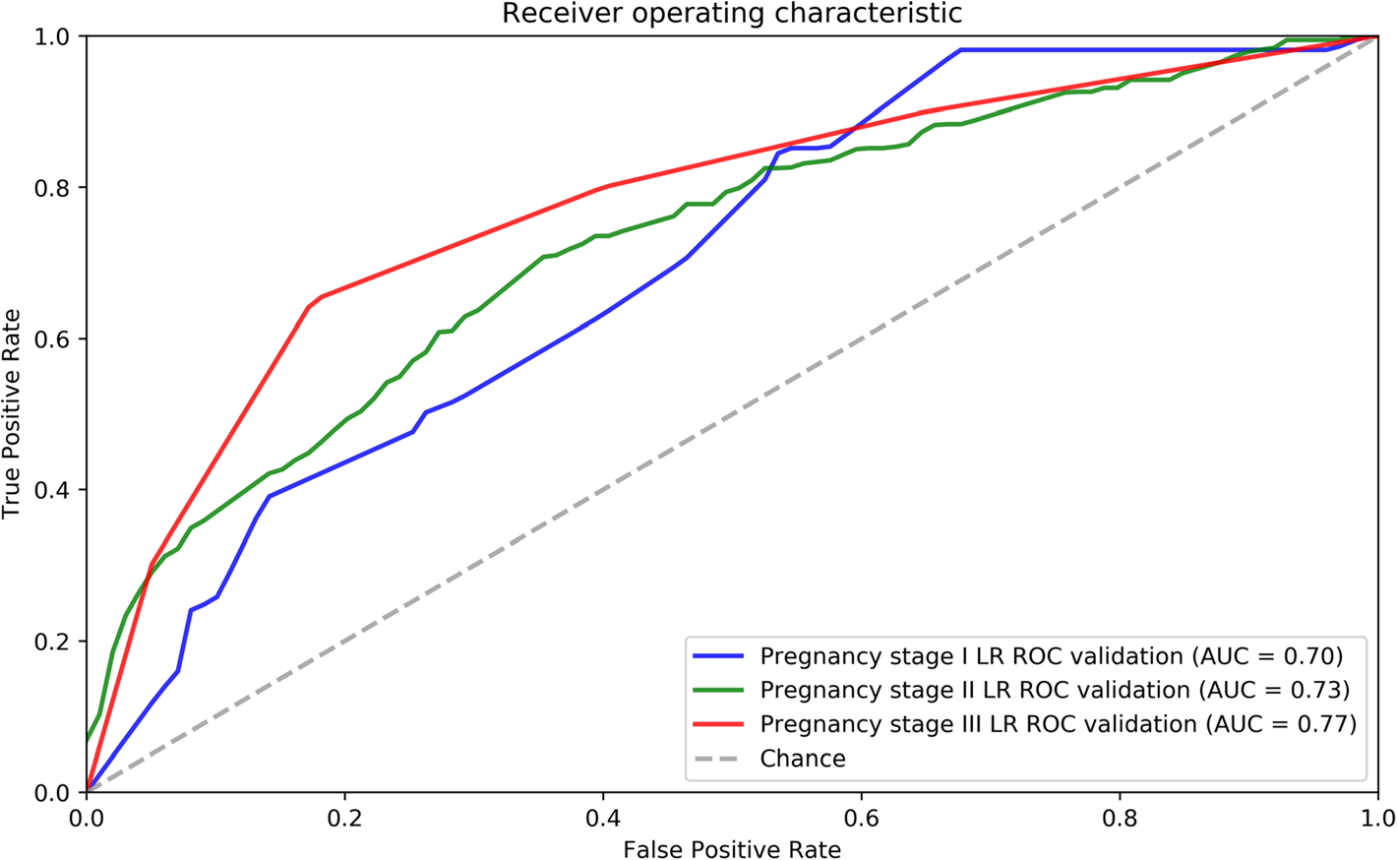
Logistic regression models of monitoring UIC among pregnancy women of the three trimesters. **A** The AUC of the model of the first trimester was 0.70. **B** The AUC of the model of the second trimester was 0.73. **C** The AUC of the model of the third trimester was 0.77

Multinomial logistic regression models based on multiple variables were built to assess the risk of above adequate iodine intake (Fig. 1). Conventional risk factors: multivitamin supplement with iodine, iodized salt and dietary habits were incorporated into the model. The feature importance of variables in the models for different trimesters were shown in Supplementary Fig. 4. The area under the ROC curve (AUC) of the monitoring models of the first trimester of pregnancy (pregnancy stage I), second trimester (pregnancy stage II) and third trimester (pregnancy stage III) were 0.70, 0.73 and 0.77, respectively in the test set (*n* = 400) (Fig. 1) and AUC of models of the three trimesters of pregnancy in the train set (*n* = 470) were 0.72, 0.73 and 0.81 shown in Supplementary Fig. 5. Based on the models, the adjustable parameters like adjust edible salt or multivitamin supplements to non-iodized ones or reducing iodine-rich food consumption can be recommended to pregnant women to prevent more than adequate iodine intake. Besides, it is helpful to better understand the iodine nutrition status of local pregnant women and timely adjust the iodine intake strategy (Table 4).

**Table 4:** Clinical application of the models for predicting the risk of more than adequate iodine intake

Trimester	Score	More than adequate risk	Clinical recommendations
I	0.53 ~ 1.00	Medium	Replace iodized salt or reduce iodine-rich food intake according to the model
	0.22 ~ 0.53	Medium Low	Reduce iodine-rich food intake according to the model
	0.00 ~ 0.22	Low	Monitor UIC
II	0.56 ~ 1.00	Medium High	Do not eat iodine contained supplements and reduce iodine-rich food intake according to the model
	0.20 ~ 0.56	Medium Low	Reduce iodine-rich food intake according to the model
	0.00 ~ 0.20	Low	Monitor UIC
III	0.58 ~ 1.00	Medium High	Don’t eat iodine contained supplements
	0.00 ~ 0.58	Low	Monitor UIC

## Discussion

Maternal iodine status is important for the growth and development of fetus. Severe iodine deficiency or iodine excess is related to adverse fetus outcomes [16]. The World Health Organization (WHO) currently recommends using UIC from spot urine samples to describe the iodine status of a population. Despite the WHO recommendation, the sampling is convenient. 24-h urinary iodine excretion (UIE) was used as a reference standard for estimation of iodine intake. In our follow-up of more than a year, urine volume was affected by climate and drinking water, and fluctuated greatly. Therefore, we consider the operability and convenience of collection, and used spot urine to evaluate iodine nutritional status. The WHO recommends that the median UIC for pregnant women is 150–249 μg/L [17]. This study showed that the median UIC for pregnant women was 172 ± 135 μg/L (sufficient by WHO criteria) which was similar compared with previous study which included 2087 pregnant women in Wuhan City, another city in Hubei Province and found that the average UIC was 178 μg/L. Universal salt iodization is the first-line strategy for the elimination of severe iodine deficiency. After the implementation of a USI policy [18], China has nearly wiped out IDD in the past two decades. In addition, increasing dietary iodine intake is an important manner to prevent iodine deficiency. Authorities recommend that pregnant women should supplement 150 mg iodine daily to achieve a total daily iodine intake of 250 mg [19]. Here, we found significant associations between iodine contained supplements and UIC though only 14.3% of pregnant women in this cohort took iodine contained supplements. What’s more, it comes from multivitamin (containing iodine) supplements. A positive significant correlation between UIC and more frequent milk consumption was found which was consistent with previous studies [20–23]. Seafood is still the main food source of iodine and positively correlated with UIC in vivo. A study conducted in Korea showed a significant correlation between dietary iodine in seaweed and UIC [24]. However, in central China, seafood is not the main diet for residents, and only 15.5% of the subjects in this study consumed seafood frequently, so there was no significant correlation between seafood consumption and UIC. In this cohort, 90.7% of pregnant women used iodized salt, which was associated with higher UIC compared to pregnant women not taking iodized salt. According to the urinary iodine level of Chinese residents, the amount of iodine in iodized salt is always changing. In 2011, the Ministry of Health of the People’s Republic of China issued the “iodine content of edible salt”, which stipulates that the average level of iodine content in edible salt can be divided into 20 mg/kg, 25 mg/kg and 30 mg/kg. One or two kinds of iodized salt can be selected according to the iodine nutrition level of people in the province in China. Xiangyang and Pingdingshan municipal government both chose 25 mg/kg standard. We confirmed the amount of iodization through the salt package provided by pregnant women. But still not sure if this dose is appropriate for each individual. Clinical factors, such as multivitamin supplements containing iodine and dietary intake are considered risk factors for iodine excess. We recalculated odds ratio (OR) value in multivariable logistic regression model, and found that two variables, multivitamin supplements with iodine and frequent milk consumption, with OR greater than 1 and P less than 0.05, were regarded as risk factors for more than adequate iodine (UIC ≥ 250 μg/L) (Supplementary Table 2). However, they are not independent risk factor. However, the increase of thyroid diseases related to excessive iodized salt has become a new public health problem [25–27]. The harm of iodine excess to pregnant women and fetus, the recommended safe dose of iodine intake is controversial and, especially for women with mild to moderate iodine deficiency. Routine monitoring is necessary to guarantee adequate iodine status. Therefore, the present study aimed to assess iodine status in central China and explore the effect of maternal UIC on neonatal outcomes. Because the best indicator to evaluate iodine status is still controversial.

Infants with maternal UIC below 150 μg/L had lower birthweight, shorter femur length and smaller head circumference than those with maternal UIC between 250 and 449 μg/L in our study. Otherwise, this study demonstrated that neonatal features were associated with dairy food and supplements. Iodine supplement, seafood, milk, yogurt intake and iodized salt consumption were positively associated with birthweight. This finding above was consistent with previous review which suggested the associations between iodized salt consumption and the increase of birthweight [28, 29]. Significant difference was also found in birthweight among different UIC groups (the birthweight in the UIC ≥ 250 μg/L group was heavier). More macrosomia occurred in pregnant women with more than adequate iodine than in those with iodine deficiency (52.8% vs. 16.7%, *P* = 0.001). Then multivariate logistic regression analysis was used to evaluate whether UIC was the risk factor for macrosomia risk. The result showed that more than adequate (UIC ≥ 250 μg/L) and iodine excess (UIC ≥ 500 μg/L) during pregnancy acted as risk factors for macrosomia. Therefore, for pregnant women in central China who generally ate iodized salt, they need to take iodine-containing multivitamin carefully. No correlation between UIC during the third trimester and first trimester of pregnancy and macrosomia was observed. High thyroid-stimulating hormone (TSH) (TSH > 4.94 nIU/l) or low free thyroxine (FT4) (FT4 < 9.01 pmol / L) had no adverse effect on macrosomia. Iodine deficiency was also not a risk factor for macrosomia (Supplementary Table 4). To monitor iodine status to prevent the risk of more than adequate iodine intake in pregnant women with normal UIC at present, thus avoiding the risk of adverse pregnancy outcomes such as macrosomia, the clinical model is established to monitor the iodine nutritional status of pregnant women in the central China to avoid more than adequate iodine intake, thus reducing the risk of having a macrosomia. Because the random urine iodine test can only represent the iodine level of this time and cannot fully reflect the iodine status in the body, nor can it show that iodized salt or iodine-rich diet intake is suitable, so how to predict the high-risk group of pregnant women with more than adequate iodine through multiple variables is meaningful, especial for pregnant women in central China who generally eat iodized salt, they need to take iodine-containing multivitamin or food carefully. In the machine learning framework, in brief, this is an online model. The model is based on the data of 870 pregnant women. The intake of iodized salt and the frequency of milk and other iodine-rich diet was taken as variables and input into the model, which have different important values (Supplementary Fig. 4). Then they will get a prediction score, which indicates the iodine nutritional status of the pregnant women, especially the pregnant women whose UIC showed that iodine was adequate can avoid iodine excess via adjustment measures given by the model. We believe that the combination of multiple risk factors is more effective in predicting risk than single indicator such as random spot urinary iodine or 24-h UIE. Then clinical guidance such as replacing iodine contained salt or supplements to non-iodine ones or reducing iodine-rich food can be output according to the risk range, and the frequency of reduction can be output, thus helping clinicians to provide prevention for more than adequate iodine intake in the pregnant women in different pregnant time. For pregnant women whose UIC has exceeded the adequate range, they can cooperate with the UIC value to evaluate the iodine-rich diet of pregnant women and give adjustment measures. The sample was relatively small in this study, thus a large population in the further studies will be analyzed to verify the role of the monitor models in clinical practice.

## Conclusions

Iodine is currently considered to be sufficient in the central China. But it is necessary to publicize the harm of iodine-excess, and its potential risk of macrosomia. The clinical model of each trimester of pregnancy was established to minimize the risk of more than adequate iodine intake, thus reducing the risk of adverse pregnancy outcomes. In addition, it is helpful to better understand the iodine nutrition status of local pregnant women and timely adjust the iodine intake strategy.

## Supplementary Information

**Additional file 1.**

## Data Availability

The datasets used and/or analysed during the current study available from the corresponding author on reasonable request.

## Abbreviations

UIC: Urinary iodine concentration
TH: Thyroid hormones
WHO: World Health Organization
USI: Universal salt iodization
IDD: Iodine deficiency disease
BMI: Body mass index
SD: Standard deviation
ROC: Receiver operating characteristic
AUC: Area under the curve
TSH: thyroid-stimulating hormone
FT4: Low free thyroxine OR Odds ratio
CI: Confidence interval
