# Supplementary material for: Iodine monitoring models contribute to avoid adverse birth outcomes related more than adequate iodine intake
Source: BMC Pregnancy Childbirth. 2021 Jun 28;21:454. doi: 10.1186/s12884-021-03936-w (PMC8240367; doi:10.1186/s12884-021-03936-w)
Supplement: Supplementary file 1 — Additional file 1. [file 12884_2021_3936_MOESM1_ESM.docx]

Supplementary Table 1. Association between UIC and clinical characteristics

|  | UIC (μg/L)  (mean ± SD) | *P*-value |
| --- | --- | --- |
| Age, years |  |  |
| 0-25 | 208 ± 137 | 0.928 |
| 26~35 | 204 ± 133 |  |
| >35 | 194 ± 158 |  |
| Gestational week |  | 0.005^*^ |
| first trimester | 194 ± 115 |  |
| second trimester | 208 ± 140 |  |
| third trimester | 200 ± 142 |  |
| Pre-pregnancy BMI, kg/m^2^ |  | 0.638 |
| <18.5 | 205 ± 119 |  |
| 18.5~24 | 206 ± 134 |  |
| 24.1~28.9 | 199 ± 142 |  |
| ≥29 | 208 ± 147 |  |
| Multivitamin with iodine |  | <0.0001^*^ |
| Yes | 321 ± 147 |  |
| No | 149 ± 84 |  |
| Sea food (fish, crab, shrimp) |  | 0.218 |
| Frequent | 224 ± 160 |  |
| Occasional | 203 ± 129 |  |
| None | 185 ± 139 |  |
| Egg |  | 0.887 |
| Frequent | 205 ± 141 |  |
| Occasional | 204 ± 129 |  |
| None | 210 ± 161 |  |
| Cow’ milk |  | <0.0001^*^ |
| Frequent | 233 ± 144 |  |
| Occasional | 188 ± 124 |  |
| None | 122 ± 86 |  |
| Yogurt |  | 0.966 |
| Frequent | 212 ± 150 |  |
| Occasional | 205 ± 136 |  |
| None | 198 ± 122 |  |
| Iodized salt |  | <0.0001^*^ |
| Yes | 222 ± 130 |  |
| No | 36 ± 27 |  |

*Statistically significant difference set at *P* < 0.05, analysis of variance (ANOVA).

Supplementary Table 2. Multivariable logistic regression analysis of risk factors for more than adequate iodine intake

| Variables | OR value | 95.0% CI for β | *P*-value |
| --- | --- | --- | --- |
| Age | 0.98 | 0.94-1.02 | 0.378 |
| Trimester | 0.89 | 0.64-1.23 | 0.496 |
| Multivitamin with iodine | 7.37 | 5.28-10.39 | <0.001 |
| Iodized salt | 9.78 | 4.82-12.89 | 0.970 |
| Sea food | 1.01 | 0.71-1.44 | 0.956 |
| Cow’s milk | 1.53 | 1.13-2.08 | 0.007 |
| Yogurt | 1.01 | 0.77-1.32 | 0.938 |
| Egg | 0.88 | 0.64-1.20 | 0.427 |

OR, odds ratio; CI, confidence interval.

Supplementary Table 3. Neonatal characteristics according to different maternal UIC

|  | UIC (μg/L) | | | |
| --- | --- | --- | --- | --- |
|  | 0-149 | 150-249 | 250-499 | ≥500 |
| Birth height (cm) | 49.9±1.6^a^ | 50.0±1.7^a^ | 50.0±1.6^a^ | 50.0±2.3^a^ |
| Birth weight (g) | 3309.1±274.8^a^ | 3336.3±283.4^a^ | 3547.7±269.0^b^ | 3518.2±265.9^b^ |
| Femur length (cm) | 7.3±0.3^a^ | 7.3±0.3^a,b^ | 7.4±0.9^b^ | 7.3±0.3^a,b^ |
| Head circumference (cm) | 33.1±1.1^a^ | 34.0±1.3^b,c,d^ | 33.8±1.3^c,d^ | 33.6±1.3^d^ |

Data presented as mean±SD.

^a^, ^b^, ^c^, ^d^ indicated statistically significant difference at P <0.05, Kruskal-Wallis H test.

Supplementary Table 4. Multivariate logistic regression analysis on macrosomia risk

| UIC (μg/L) | Macrosomia  OR (95% CI) | *P*-value |
| --- | --- | --- |
| ≥250 | Ref |  |
| 0-149 | 2.51 (1.33, 7.54) | 0.097 |
| 150-249 | 1.41 (0.61, 3.97) | 0.345 |

Data presented as odds ratio (OR) (95% confidence interval (CI)).

*Statistically significant difference set at P <0.05.


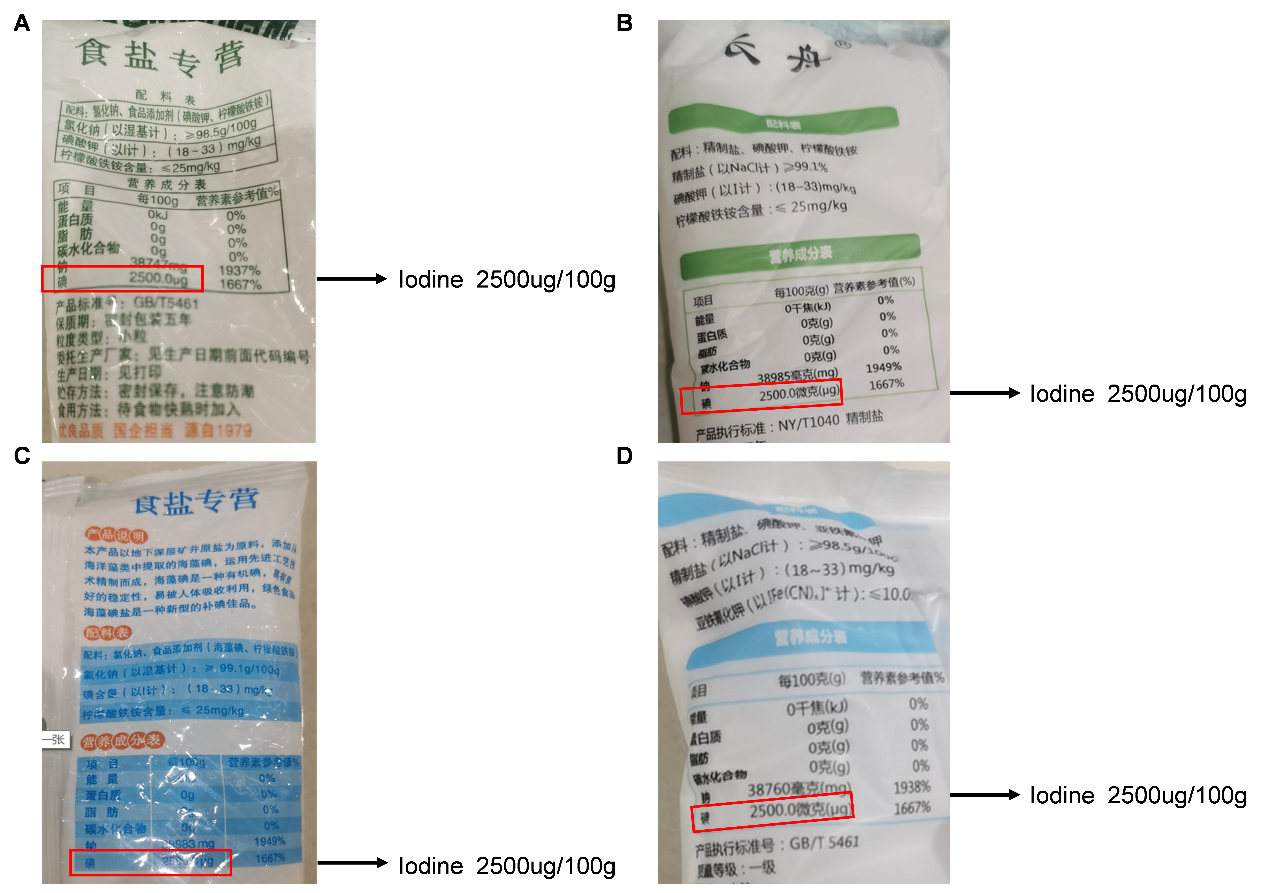


Supplementary Figure 1. The photo templates of the package of household salt.


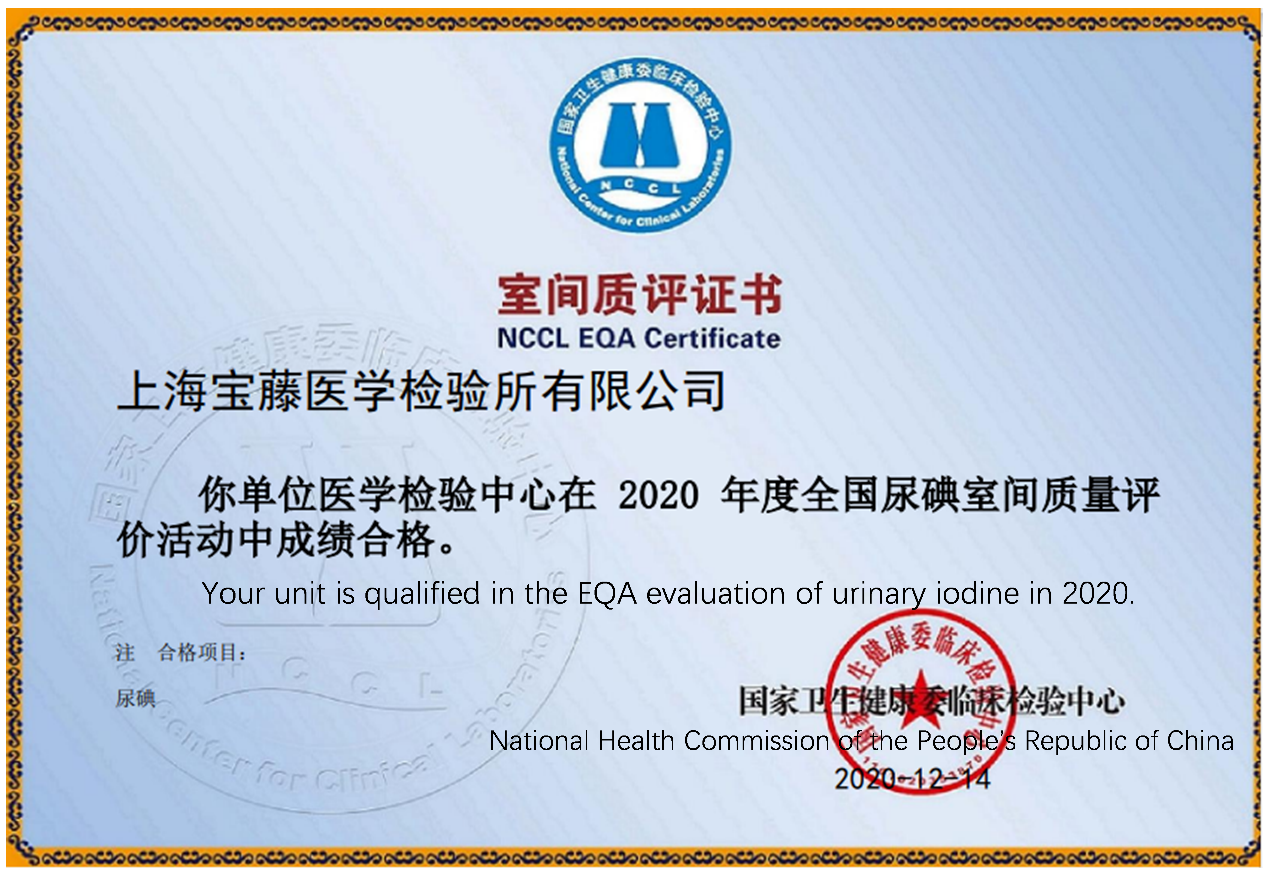


Supplementary Figure 2. A recent “Successful” participation certificate of EQA issued by the National Health Commission for the ICP/MS method in December 2020.


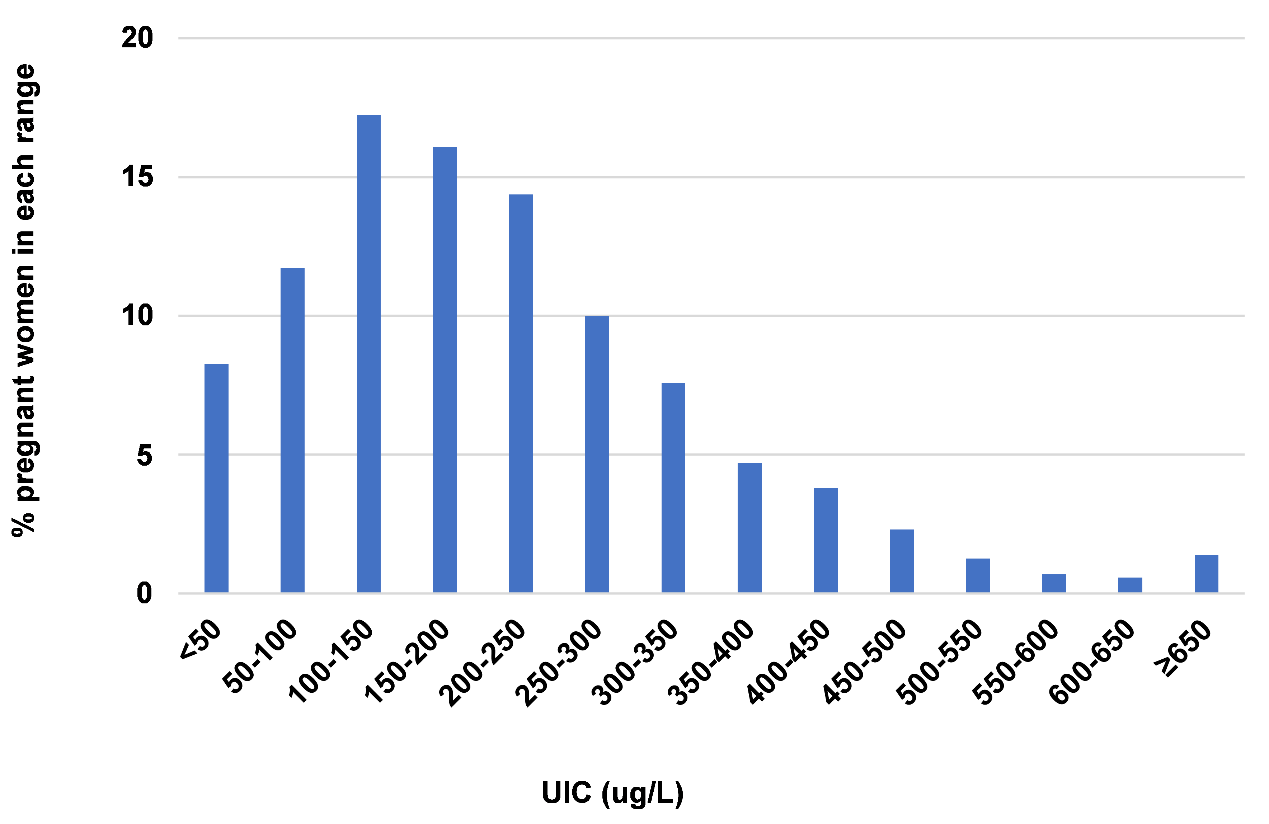


Supplementary Figure 3. Distribution of UIC in total pregnant women.

Supplementary Figure 4. The feature importance of variables in the model of the first trimester
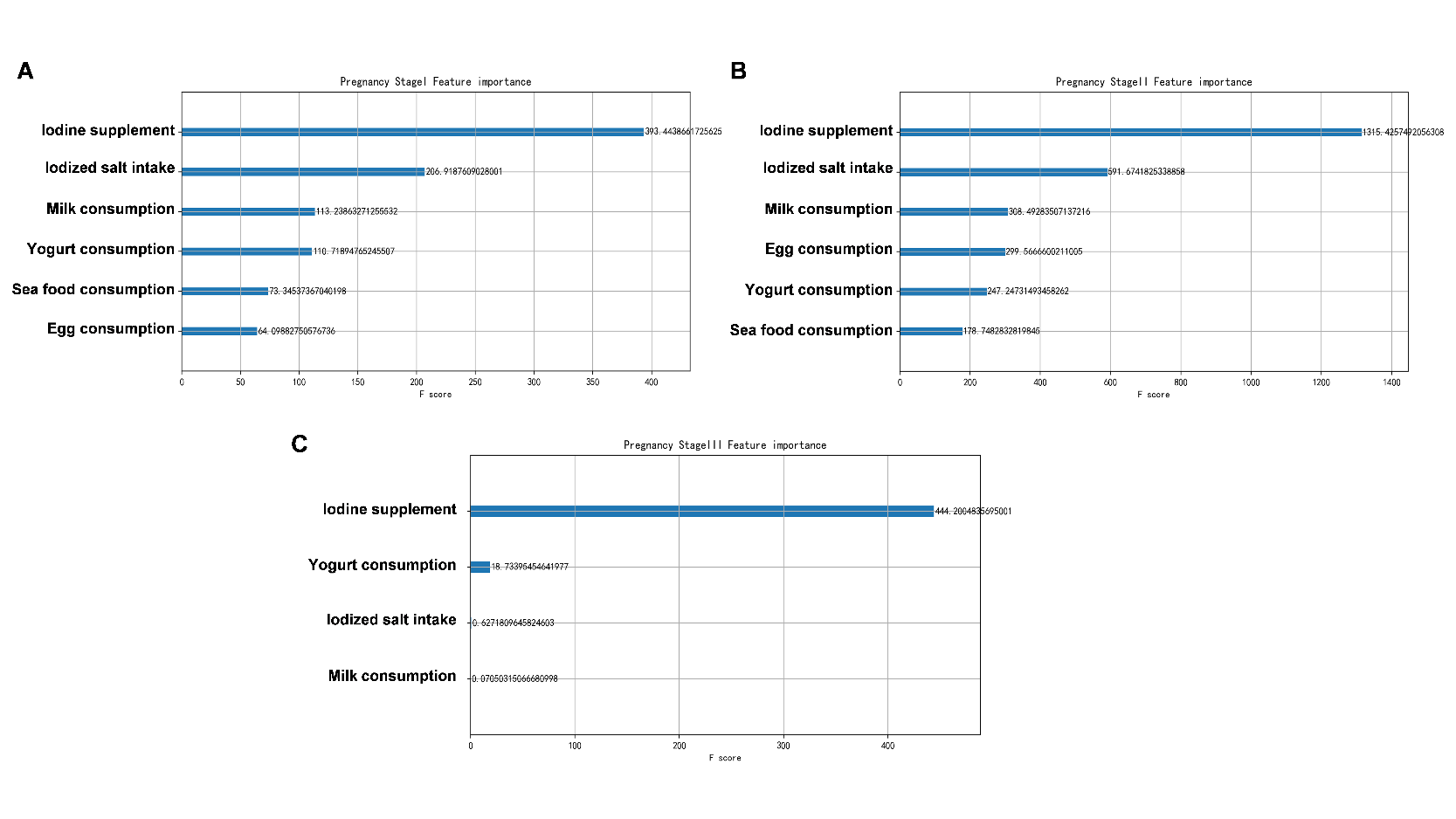
 of pregnancy (A), the second trimester of pregnancy (B) and the third trimester of pregnancy (C).


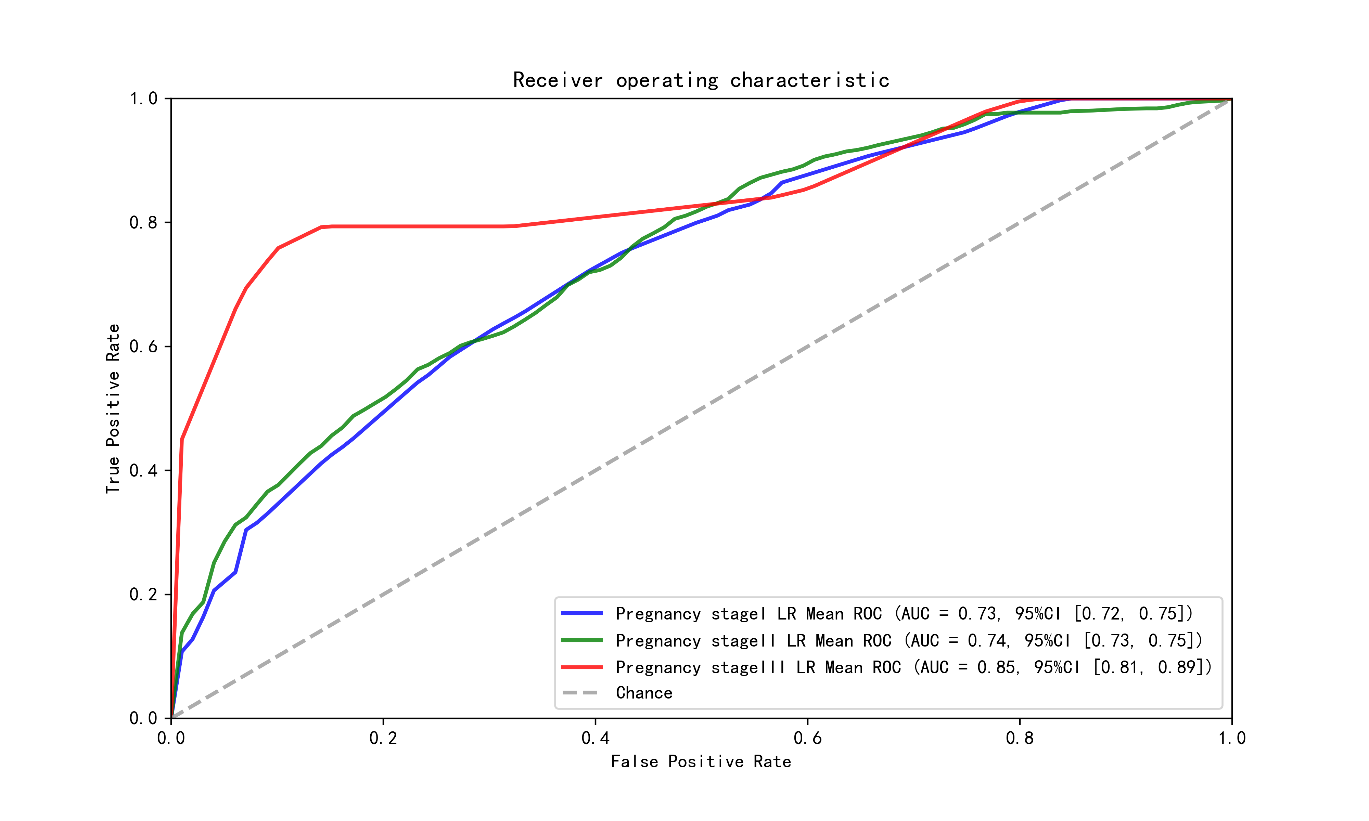


Supplementary Figure 5. The area under the curve (AUC) of models of the three trimesters of pregnancy in the train set were 0.72, 0.73 and 0.81, respectively.
